# Assessing contribution of bottled water in nutrient absorption using the bottled water nutritional quality index (BWNQI) in Iran

**DOI:** 10.1038/s41598-021-03792-w

**Published:** 2021-12-21

**Authors:** Masoomeh Askari, Reza Saeedi, Ramin Nabizadeh, Ahmad Zarei, Maryam Ghani, Marzieh Ehsani, Mahmood Alimohammadi, Mehrnoosh Abtahi

**Affiliations:** 1 Master Student of MPH, Department of Environmental Health Engineering, School of Public Health and Safety, Shahid Beheshti University of Medical Sciences, Tehran, Iran; 2 Workplace Health Promotion Research Center, Shahid Beheshti University of Medical Sciences, Tehran, Iran; 3 Department of Health, Safety and Environment, School of Public Health and Safety, Shahid Beheshti University of Medical Sciences, Tehran, Iran; 4 Department of Environmental Health Engineering, School of Public Health, Tehran University of Medical Sciences, Tehran, Iran; 5 Department of Environmental Health Engineering, School of Public Health, Infectious Diseases Research Center, Gonabad University of Medical Sciences, Gonabad, Iran; 6 Dentistry Student, School of Dentistry, Tehran University of Medical Sciences, Tehran, Iran; 7 Center for Water Quality Research (CWQI), Institute for Environmental Research (IER), Tehran University of Medical Sciences, Tehran, Iran; 8 Health Equity Research Center (HERC), Tehran University of Medical Sciences, Tehran, Iran; 9 Department of Environmental Health Engineering, School of Public Health and Safety, Shahid Beheshti University of Medical Sciences, Tehran, Iran

**Keywords:** Environmental sciences, Hydrology

## Abstract

In this study, the contribution of bottled water in the absorption of nutritional minerals in Iran has been investigated. To calculate the nutritional quality index of bottled water (BWNQI) and evaluate the contribution of bottled water in nutrient absorption; the concentration of nutrient minerals, the standard level of these elements in bottled water, the recommended amount of nutrient mineral and the total consumption of drinking water in different age-sex groups were analyzed. The results showed that the average contribution of bottled water in absorbing the recommended amount of the nutrients of fluoride (F), magnesium (Mg), calcium (Ca), sodium (Na), copper (Cu), zinc (Zn) and manganese (Mn) was 12.16, 4.98, 4.85, 2.12, 0.49, 0.33 and 0.02%, respectively. According to the BWNQI index, the bottled water quality was as follows: 53.5% poor, 36.6% marginal, 7% fair, 2.81% good. Although most of the bottled water studied in this research were mineral water, a significant portion of them had poor nutritional quality, so the addition of minerals needed by the body through bottled water should be given more attention by the bottled water manufacturers and suppliers.

## Introduction

Safe drinking water is vital for human health. Rapid population growth and industrialization had polluted many water resources around the world^1–3^. Recently, consumption of bottled water has increased globally. In Iran similar to other countries, the consumption of bottled water has increased due to dry and semi-arid climatic conditions and the problems of severe water shortages. Bottled water, one of the main sources of drinking water, is increasingly used in many nations especially in arid and semi-arid countries^4–6^. In many nations, drinking water is usually supplied from tap water and bottled water. There are different opinions about which water is suitable for drinking: Some people believe that tap water is not safe and does not taste good and is dangerous to health^7^. One of their reasons is the tap water enters the distribution pipes through the pumping system and may contain corrosion and dissolved materials originated from metal pipes.

Many developed countries add chlorine to tap water for disinfection. The presence of organic matters with chlorine can produce compounds which are carcinogenic for example trihalomethanes (THMs) and haloacetic acids (HAAs)^5,8^. The World Health Organization (WHO) recommends a chlorine concentration range of 0.2–0.5 mg/L in drinking water and the remaining chlorine at 0.2 mg/L to protect the health of the consumer. Many consumers do not consume tap water because they think it is not safe. Some consumers also believe that tap water is healthier, more environmentally friendly, and cheaper than bottled water. Some people believe that bottled water is actually tap water^9–11^. The sources of bottled water are from spring, boreholes, tubular wells water and urban distribution water systems. These resources must be safe if used for human consumption^7^.

In addition to people's tastes, due to changing lifestyles and their tendency towards a healthy lifestyle and not only quenching their thirst, today different brands of bottled water are used around the world^12^. Demand for bottled water in North America has shown an annual growth rate of 25%. The annual bottled water consumption was reported 45 L/year per capita in 2003 in Europe^13^. Globally, the use of bottled water has obeyed a continuously increasing trend in recent 14 years. For example in Japan, the amount of water consumption was reported 31.7 L/year per capita in 2019^14^.

Global consumption of bottled water in 2013 was 70,371.6 million gallons which was 6.2% more than in 2008^11^. Iran is also facing a severe shortage of water due to dry and semi-arid climates in many provinces. The amount of renewable water in Iran is less than 2000 cubic meters per person per year, which according to the current population growth, is expected to reach less than 1000 cubic meters per year by 2025. Therefore, it is necessary to use alternative water resources to meet the country's water needs^15^.

Bottled water contains cations such as sodium (Na), calcium (Ca), magnesium (Mg), and potassium (K), and anions such as sulfate (SO_4_), chloride (Cl^-^), and bicarbonate (HCO_3_). They have good quality due to the special characteristics of the taste and content of various mineral compounds^16^. Generally, bottled water should have a high concentration of Ca and Mg and a low concentration of Na^17^. Some of the minerals in bottled water, including Ca, Cl^-^, Cu, F, I, Fe, Mg, Mn, P, K, Se, Na, Zn, etc., are among the nutrients necessary for the human body^18^. Elevated concentrations of these minerals can reduce the quality of bottled water and be harmful to human health. According to the standard of bottled drinking water in Iran, the maximum allowable amount of Ca, Mg, Na, Mn, Cu, Zn and F minerals is 40–80, 20–30, 200, 0.5, 1, 0.15 and 4 mg/L, respectively^3^. Some studies show that many of the minerals in bottled water can have negative effects on human health through toxic mechanisms^19^. For example, Cu at concentrations above 100 mg/L in the human body can lead to the destruction of the oxidative cell and eventually death^20^. Absorption of more than 1000 mg per day of Ca in the body can lead to kidney stones^21^. There are also concerns about high concentrations of F in bottled water^22–24^. High Na concentration raises blood pressure^25,26^. Mn and Zn minerals, which are in the heavy metals category, accumulate in high concentrations after entering the body in fat, muscle and bone, and generally cause neurological disorders, cancer and, in acute cases, death^27,28^. In addition to the harmful effects, some minerals have beneficial effects on human health. According to epidemiological studies, Mg can reduce sudden death and prevent Ca from osteoporosis^17,29^.

Studies have shown that proper nutrition is effective in absorbing essential minerals in the body, and drinking water has also an important role in the absorption of minerals^19,29,30^. Sajjala et al. studied several brands of bottled water in Oman in 2019. They found that the concentration of Ca in local brands was low compared to the imported brands and the contribution of this element for the recommended dietary allowances of Ca in adults was only 3% and the contribution of Na was also very low^29^. In another study in 2020, Cormick et al. reported that most of bottled water brands in Argentina had Ca levels below 50 mg/L. According to their study, the intake of 1 L of drinking water from Argentina could represent in averagely 1.2–8 percent of the Ca daily levels for an adult person^31^. Duka et al. in a study found that dietary reference intakes of Ca and Mg minerals from bottled water was averagely 8.29 and 4.27%, respectively in commercial bottled drinking water in Tirana, Albania During 2019^32^.

There are few studies in Iran regarding the contribution of bottled water in the intake of nutrients, therefore the study of nutritional quality of the bottled water in necessary. Herein, the aim of this study was to evaluate the contribution of bottled water in the absorption of Ca, Cu, F, Mg, Mn, Na, and Zn minerals in different age-sex groups and in the general population using bottled water quality food index (BWNQI).

## Results and discussion

### The concentration of mineral nutrients

The concentration of minerals in bottled water in 71 brands was shown in Table 1.

**Table 1:** The concentration of minerals in bottled water in 71 studied brands.

Brand /Nutrients (µg/L)	Ca	Cu	F	Mg	Mn	Na	Zn
1	63100	7.867	400	16.67	0.05	11990	5
2	4500	25.988	198	1.512	0.05	13075	5
3	57275.5	5.449	1553	10.8545	0.05	2447.5	3
4	45929	3.929	230	4.858	0.05	11280	2
5	35960	3.802	600	6.101	0.05	1230	4
6	16703	3.896	41	2.613	0.05	10841	1
7	74259.5	3.271	552	18.9125	0.05	12086	1
8	14588.5	3.599	370.5	3.5485	0.05	27818	1
9	43738	2.298	790	12.207	0.05	21460	10
10	20519	2.658	27	8.732	0.05	16650	18
11	25111	2.183	118.5	17.017	0.05	2563.5	6
12	20060	2.199	269	5.282	0.05	38105.5	0.29
13	20373	1.876	431	5.9295	0.05	21091.5	1
14	47012	3.053	240	4.978	0.05	1540	7
15	22376	2.67	160	2.921	0.05	20000	3
16	26955.5	2.059	86.5	4.9745	0.05	15281	17
17	15989	2.16	588	2.6345	0.05	101704	8
18	40436.5	2.22	236.5	5.641	0.05	6721	1
19	37669.5	1.967	365	8.094	0.05	12487.5	2
20	52542.5	1.83	233.5	7.627	0.05	12258.5	3
21	37337.5	2.37	100	4.9025	0.05	6312	1
22	33192	2.252	105	5.1605	0.05	61079	2
23	55708	1.411	326.5	9.3735	0.05	10458.5	7
24	14125	1.611	213.5	2.501	0.05	26897	21
25	13553	2.187	82.5	2.8125	0.05	26931	14
26	36688.5	2.176	272	14.821	0.05	22847	7
27	22500.5	2.067	74	6.072	0.05	17585.5	3
28	42460	1.419	135.5	9.7545	0.05	3064.5	35
29	35668	1.625	580	12.181	0.05	11870	16
30	45913	2.344	142	12.85	0.05	19771	3
31	38103	1.715	240	8.721	0.05	7310	2
32	30246.5	2.817	396.5	11.1545	0.05	14609.5	1
33	48715	1.708	486	12.9335	0.05	9722.5	6
34	68924.5	1.922	556.5	15.759	0.05	4822	28
35	55454.5	1.589	301	13.731	0.05	17249	6
36	78238.5	2.63	191	5.975	0.05	9224	4
37	47712.5	1.363	256.5	11.7205	0.05	2043.5	46
38	28233.5	2.038	305	4.871	0.05	16331	7
39	55140	1.629	281	6.582	0.05	9860.5	36
40	32600.5	2.125	415.5	6.497	0.05	31433.5	13
41	36713.5	1.383	526	6.712	0.05	29942	26
42	64359	0.808	272	15.8275	0.05	4867.5	307
43	36922.5	0.743	155.5	9.3425	0.05	24922	25
44	21832	19.708	95	7.522	0.05	15152	9
45	20211	0.33	33	6.194	0.05	32696	21.427
46	27899	0.689	408	17.022	0.05	58685	9.097
47	23950	2.904	150	10.41	0.05	19320	1.346
48	23349	0.499	154	4.615	0.05	28620	55.582
49	27996	0.33	272	8.807	0.05	21600	1.065
50	51863	0.33	389	10.115	0.05	31269	11.375
51	87837	0.33	207	9.788	0.05	9699	56.185
52	69922	0.33	272	19.326	0.05	98506	10.53
53	44565	0.33	119	9.16	0.05	29528	55.359
54	41614	1.145	973	6.06	0.05	161000	10.658
55	19636	0.33	108	1.875	0.05	44817	6.976
56	27361	0.559	27	6.86	0.05	2738	5.255
57	46927	0.33	237	4.484	0.05	58241	0.474
58	59838	0.33	307	8.887	0.05	20014	0.326
59	59008	0.33	100	12	0.05	4864	59.063
60	40584	0.33	82	9.695	0.05	5422	30.26
61	41450	0.33	473	7.145	0.05	56004	8.01
62	80155	0.33	200	10.721	0.05	5140	3.361
63	45142	0.33	84	6.216	0.05	2656	6.648
64	49784	0.33	276	5.795	0.05	44835	2.111
65	54694	0.33	141	9.553	0.05	5376	5.144
66	11702	0.33	46	4.402	0.05	29802	2.414
67	22928	0.33	82	6.643	0.05	5000	2.024
68	23817	57.993	50	5.392	0.05	10401	1.511
69	24863	12.038	69	6.606	0.05	16051	4.407
70	25272	5.724	43	5.791	0.05	41305	2.329
71	26500	1.628	30	5.93	0.05	22540	5.533

The maximum values of Ca, Cu, F, Mg, Mn, Na and Zn were 87837, 57.993, 1553, 19.326, 0.05, 161000 and 307 µg/L respectively. The results showed that the concentrations of all minerals in the study brands were within the maximum contaminant levels of the EPA standards^33^.

### Determination of the contribution of bottled water in the absorption of the recommended amount of nutrients by the body in the entire population

The average contribution of bottled water in absorbing the recommended amount of nutritional minerals in 71 brands of bottled water in the entire population of Iran in different age-sex groups is shown in Table 2. Based on the results, the average contribution of 71 brands of bottled water in Ca absorption in the age-sex groups of infants aged 6–12 months, children aged 1–9 years, adult men and women were 5, 3.3, 4.84 and 4.17%. respectively. The average contribution of 71 brands of bottled water in Cu absorption in the sex-age groups of infants aged 6–12 months, children aged 1–9 years, male and female adults were 0.76, 0.36, 0.5 and 0.47%, respectively. For fluoride, this mean absorption in the age-sex groups of infants aged 6–12 months, children aged 1–9 years, adult males and females was 28.8, 15.93, 12.17 and 10.38%, respectively. Furthermore, The average contribution of bottled water in Mg absorption in the age-sex groups of infants in 6–12 months, children 1–9 years old, adult men and women was 9.75, 5.44, 4.58 and 4.95%, respectively.

**Table 2:** The average contribution of bottled water in nutrient uptake (%) in 71 brands of bottled water in the entire population of Iran in different age-sex groups.

Sex-age groups	Population	Average share of bottled water in nutrient uptake (%)						
	–	Ca	Cu	F	Mg	Mn	Na	Zn
**Infants**								
0–6 m	748778	4.95	0.73		11.62		8.58	0.25
7–12 m	748778	5.12	0.79	28.82	7.89	0.0294	3.29	0.20
**Children**								
1–3 y	4239205	3.28	0.41	16.47	5.68	0.0118	0.97	0.16
4–6 y	3991675	3.51	0.41	14.82	5.77	0.0121	1.04	0.18
7–9 y	3775845	3.35	0.28	16.50	4.88	0.0126	1.07	0.17
**Females**								
10–18 y	4906221	2.70	0.33	9.86	3.32	0.0189	1.39	0.20
19–50 y	21004347	5.36	0.50	12.56	5.07	0.0256	2.12	0.44
51–65 y	4712943	4.45	0.55	13.57	5.47	0.0277	2.65	0.48
> 65 y	2235866	4.17	0.51	12.71	5.94	0.0259	2.68	0.45
**Males**								
10–18 y	5142142	2.70	0.33	9.86	3.17	0.0144	1.39	0.16
19–50 y	21554312	6.09	0.57	10.71	4.88	0.0228	2.41	0.35
51–65 y	4665812	6.06	0.57	10.66	4.85	0.0227	2.77	0.35
> 65 y	2200346	4.51	0.55	10.32	5.45	0.0220	2.91	0.34

Averagely, the contribution of bottled water in the absorption of Mn in the age-sex groups of infants aged 6–12 months, children aged 1–9 years, adult males and females was 0.029, 0.012, 0.02 and 0.024%, respectively.

Also, the average contribution of bottled water in the absorption of Na in the age-sex groups of infants aged 6–12 months, children aged 1–9 years, adult males and females was 5.93, 1.02, 2.37 and 2.21%, respectively.

The role of bottled water in the absorption of Zn in the age groups of infants aged 6–12 months, children aged 1–9 years, adult men and women was averagely 0.22, 0.17, 0.3 and 0.39%, respectively.

Adequate absorption of nutrients is usually possible through the consumption of water and food^34–36^.

In a 2019 study by Park et al., the contribution of bottled water in Ca absorption in Korean adult males and females was 3.3 and 2.9%, respectively. In the current study, adult men and women showed a higher contribution compared Park et al. study^37^.

In this study, the absorption of fluoride was higher than other nutrients. Viswanathan et al. in a study in 2010 found that daily more than 60 percent of all fluoride was absorbed through drinking water^38^. Adequate fluoride intake is effective in preventing tooth decay, but excessive and long-term use can cause fluorosis^1,39,40^. Rowell et al. in a study in Qatar found that bottled water provides 3, 5–6 and 4 percent of recommended amount of essential minerals including Ca, Mg and F in the adult body, respectively. In children, this amount was 2–3, 3–10 and 3–9%, respectively^41^. In this study, the percent of the recommended amount of essential minerals for Ca, Mg and F were higher than those reported in the Rowell’s study.

Sodium deficiency is usually rare in different age groups, but there are more concerns about increasing sodium absorption^42,43^.

The nutrient minerals studied in this work have different biological functions as Ca strengthens bones, regulates muscle contraction, blood clots, etc.^44^, Cu controls the function of several enzymes in the blood and muscles of the body^45^, Mg is involved in bone formation, lipid metabolism and protein synthesis^46^; Mn is responsible for the synthesis of several enzymes in protein and sugar metabolism and bone growth^47^. Like Mg, F is effective in bone growth and also plays an important role in protecting and preventing tooth decay; Na is a key regulator of cell permeability and body fluids^48^. Studies show that bottled waters in Europe have higher levels of minerals than in other countries. For example, about one liter of bottled water in Europe accounts for 20–58% of Ca absorption and 16–41% of Mg absorption and the highest contribution of Na absorption in adults. Bottled water in the United States generally has low mineral levels. Also, bottled water in the UAE has low mineral quality and has only a 3% contribution of Ca absorption^29,49^. In general, bottled water, which has a high mineral content, contributes 100% of the absorption of Ca, Mg and Na^34,35^.

**Figure 1:**
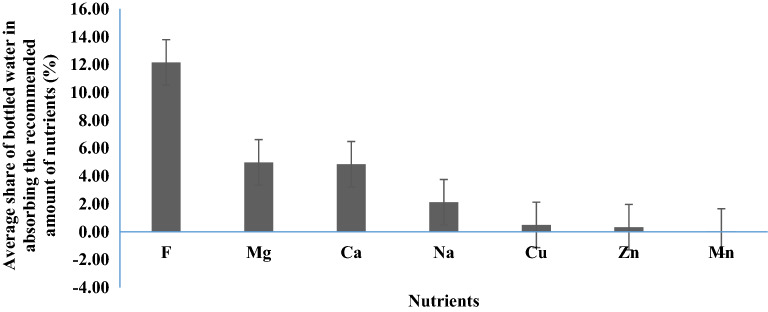
The average contribution of bottled water in absorbing the recommended amount of the nutrients of Ca, Cu, F, Mg, Mn, Na and Zn in the total population of Iran (created by: Microsoft Excel 2016, https://www.microsoft.com/en-us/microsoft-365).

The average contribution of bottled water in absorbing the recommended amount of the nutrients of Ca, Cu, F, Mg, Mn, Na and Zn in the total population of Iran was 4.85, 0.49, 12.16, 4.98, 0.02, 2.12 and 0.33% (Fig. 1).

**Figure 2:**
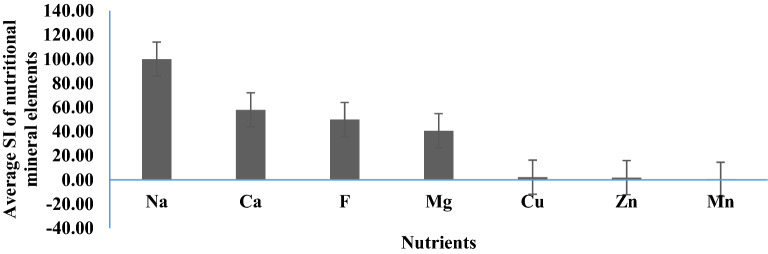
Average SI of nutritional mineral elements in different brands of bottled water (created by: Microsoft Excel 2016, https://www.microsoft.com/en-us/microsoft-365).

The mean SI scores of Ca and Mg in bottled water were 57.90 and 40.66, respectively. These results showed that Ca and Mg in bottled water did not exceed the maximum allowable amount. The mean SI score of F was 49.90 (Fig. 2).

The highest average SI score in bottled water was for Na ions, indicating that the concentration of this ion was lower than desired concentration. In general, the SI scores of Cu, Mn and Zn were much lower than other minerals.

### Determination of the quality of bottled water using the quality index of bottled water

**Figure 3:**
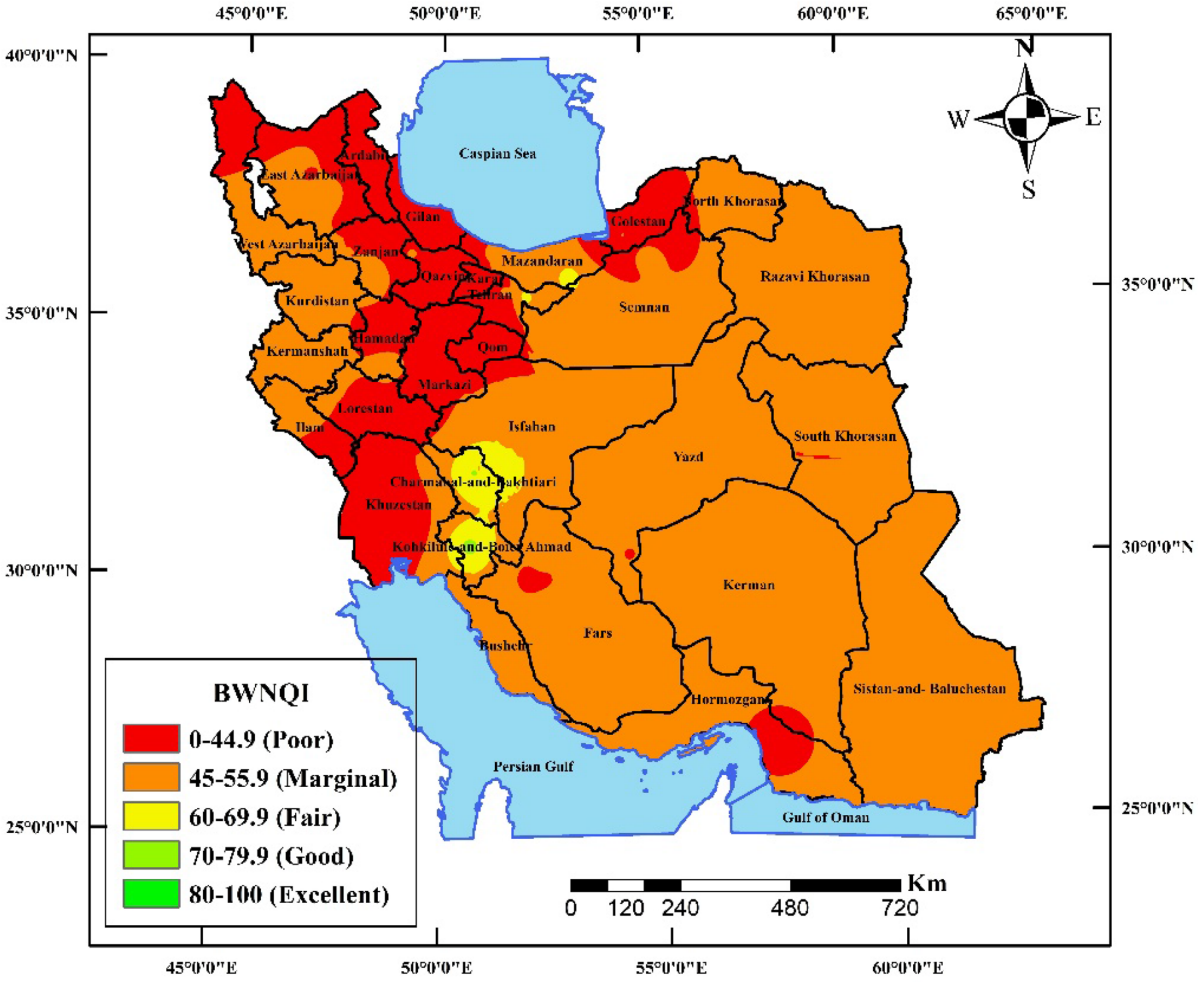
Distribution of the BWNQI score at the provincial level (ArcGIS 10.4.1 software: Environmental Systems Research Institute (ESRI) at: https://www.esri.com).

BWNQI in 71 brands of bottled water in Iran was 2.81, 7, 36.6, 53.5%, and classified as good, fair, marginal, poor quality, respectively. BWNQI index in Ardabil, Gilan, Qazvin, Karaj and Golestan provinces with score of 44.9 was in the poor range. BWNQI index in Chaharmahal and Bakhtiari and Kohkiluyeh and Boyer-Ahmad provinces with score range of 70.79–79 was in the good range (Fig. 3). More than half of bottled water in Iran have poor quality in terms of quality index. Also, BWNQI index only in two provinces in Iran was in good range.

### Relationship between bottled water source and BWNQI index

**Figure 4:**
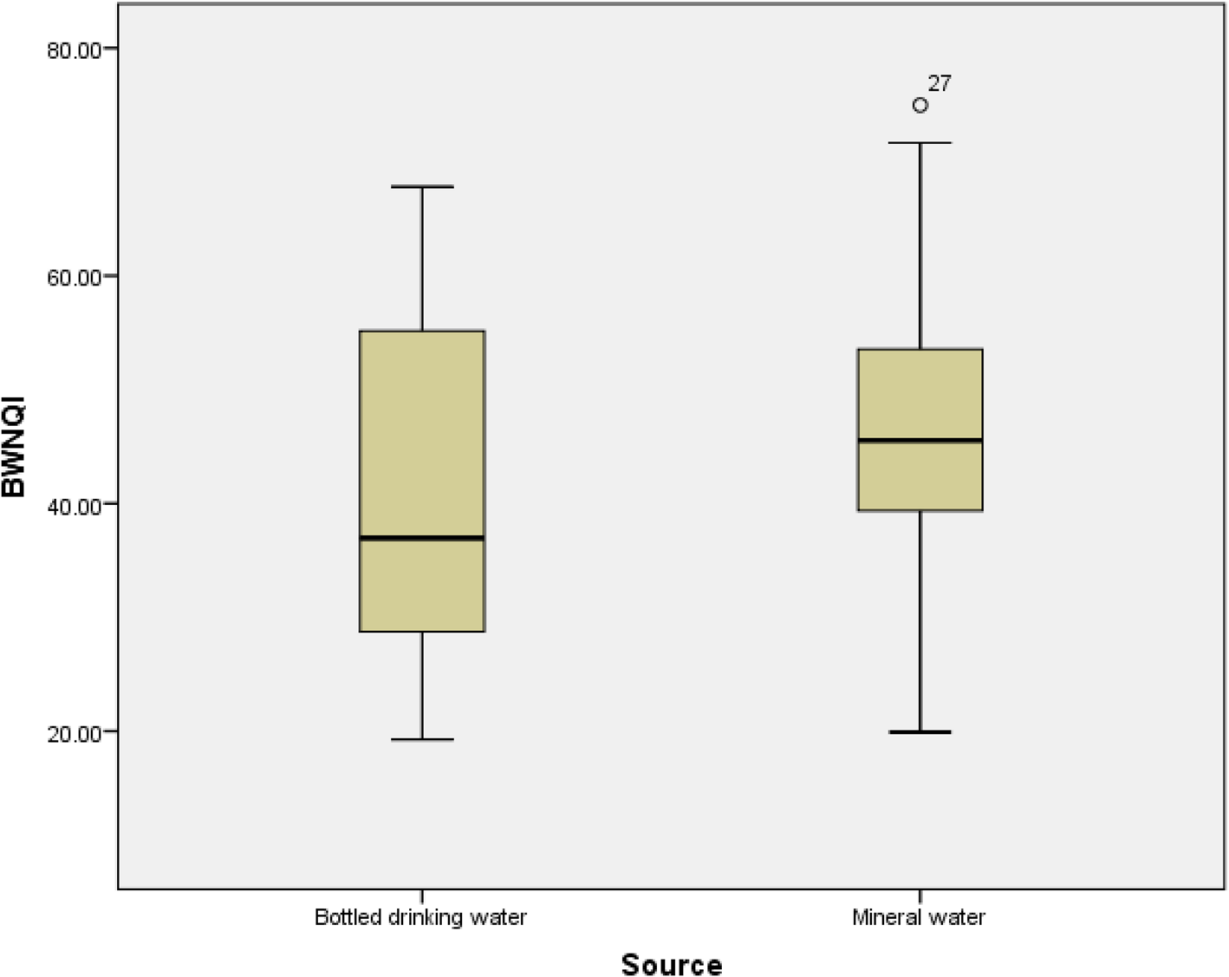
Relationship between bottled water source and BWNQI index (created by: SPSS 21.0, https://spss.software.informer.com/21.0).

The sources of bottled water in this study were bottled natural mineral water and bottled drinking water. The results of this study showed that there was no significant relationship (*P* > 0.05) between bottled water sources and BWNQI index (Fig. 4).

### Sensitivity analysis

**Figure 5:**
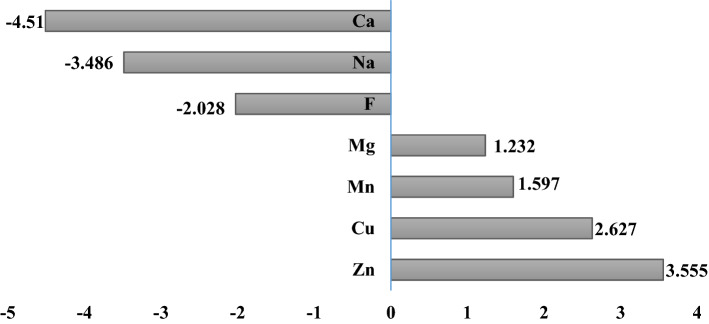
Effect of removing each input parameter on the average score of the BWNQI (created by: Microsoft Excel 2016, https://www.microsoft.com/en-us/microsoft-365).

The effect of removing each of the nutritional minerals on the average of BWNQI score is shown in Fig. 5. The effect of removal of the nutritional minerals on the average BWNAQI score was in the range of − 4.51 to 3.5. The highest positive and negative effects were related to Zn and Ca, respectively.

The average BWNQI score represents the average SI score, so the removed nutritional minerals with negative effects including Ca, Na, F had an average SI higher than the average score of BWNQI and the removed nutritional minerals with positive effects such as Mg, Mn, Cu, Zn had lower mean SI score than the BWNQI mean score.

The lowest (R^2^ > 0.8) and highest (R^2^ > 0.9) correlation coefficient with the average BWNQI score was related to F and Na, respectively. The data showed that none of the input parameters had an effect on the BWNQI score, so this index can be a good indicator to show the contribution of bottled water in nutritional minerals absorption for human body.

## Conclusions

Unfortunately, in some countries, especially in developing countries, there is no difference between drinking and bottled water. One of the reasons is that people are not aware of such waters. Due to the increasing number of such waters, people think that they receive large amounts of anions and cations. Therefore, knowing the contribution of bottled water can help the body absorb useful and essential minerals to check the percentage of essential minerals compared to their recommended amount and the role they play in people's health, as well as the nutritional quality of bottled waters are useful and effective. In this study, the contribution of bottled water consumed in Iran in the absorption of nutritious mineral elements such as calcium, fluoride, copper, manganese, zinc, magnesium and sodium was examined. According to the BWNQI index, the results of bottled water quality investigation was as follows: 53.5% poor, 36.6% marginal, 7% fair, 2.81% good. This study showed that the majority of bottled waters consumed in Iran are not good in quality and the nutrients should be added by the bottled water manufacturers based on the standards of bottled drinking water quality. Compared to nutrient minerals, fluoride absorbed more by drinking bottled water throughout Iran. Due to the increasing trend of bottled water consumption in recent years in Iran, the need for further study in the field of quality improvement and promotion of these waters and their contribution to the absorption of nutrients to the body is essential. In addition to public health issues, the nutritional value of such water should also be considered.

## Materials and methods

### Study area description

**Figure 6:**
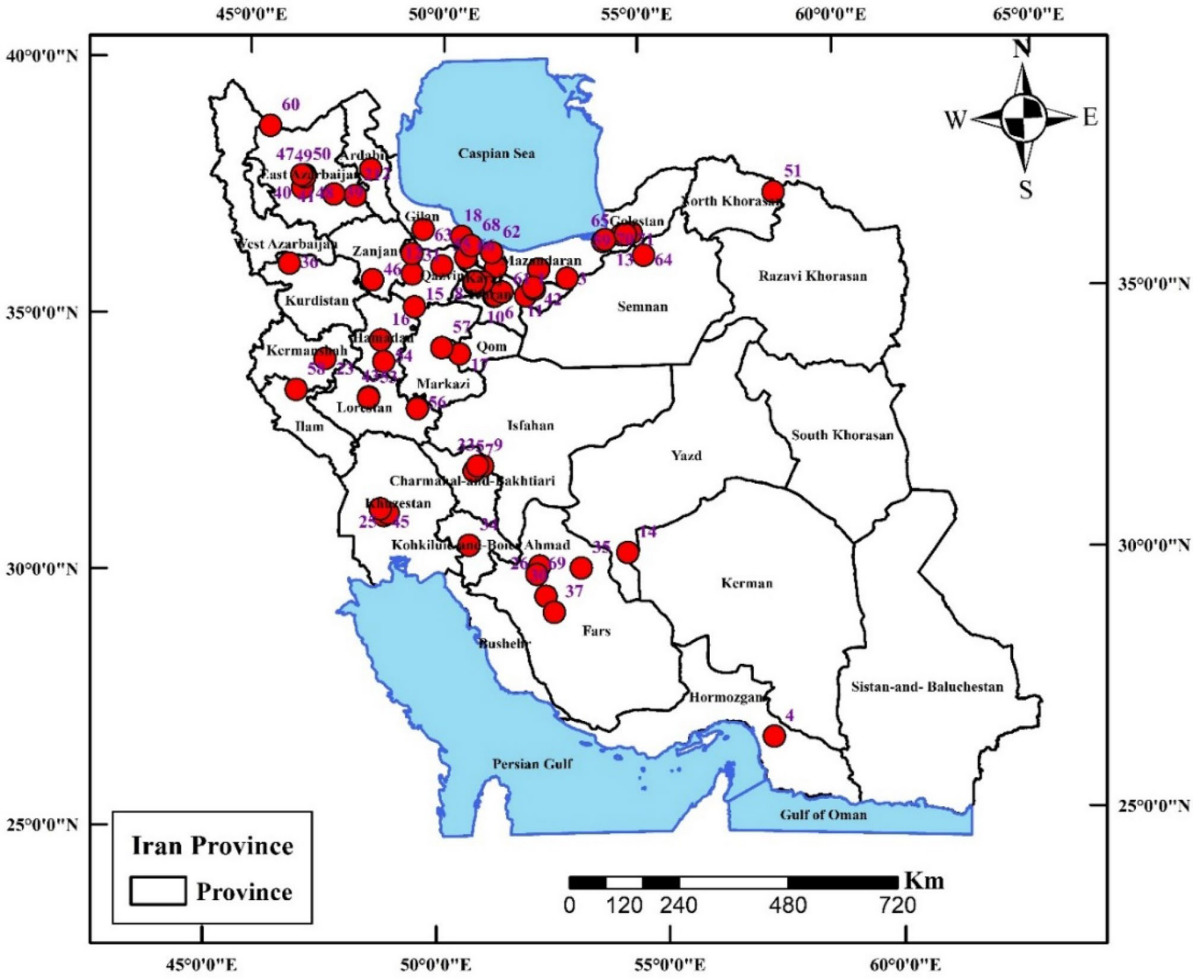
Study locations and sampling points (ArcGIS 10.4.1 software: Environmental Systems Research Institute (ESRI) at: https://www.esri.com).

This study was conducted in Iran. Iran has an area of about 1,648,195 km^2^ and a population of 85,539,819 people. The country has 32 provinces. It is located in Western Asia. About 99% of urban water supply is in the form of tap water. Given that most of the provinces are located in the dry places and the piped water tastes salty, therefore, more attention is paid toward bottled water. Study locations and sampling points are shown in Fig. 6.

### Sampling

This study was conducted randomly for 71 brands of bottled water sold in supermarkets of Iran in 2018. Three samples from each brand were collected once a week. All samples were collected in autumn.

### Sample analysis

Among all cations and anions, Ca, Cu, F, Mg, Mn, Na and Zn were selected for the purpose of this study. The collected samples did not require special preparation. These ions were analyzed according to Standard Methods for the Examination of Water and Wastewater^50^.

### Statistical analysis

For statistical analysis, SPSS software (version 21) and Excel 2016 were used. Arc GIS software (version 10.4.1) was used for spatial analysis. Spearman correlation coefficient was used to measure the relationship between bottled water sources and BWNQI index. In addition, t-test was used to quantify the statistical level of correlation coefficients.

### Instrumental analysis

The collected samples did not require any preparation before analysis. For Na, Ca, Mg, Cu, Zn and Mn analysis, an Inductively Coupled Plasma Optical Emission Spectrometer (ICP-OES) was used and F values were analyzed by an Ion Chromatography (IC- Professional Ion Chromatography 850).

### Quality assurance and quality control (QA/QC)

Analytical techniques were confirmed based on quality assurance and quality control (QA/QC). The calibration range, the concentration range and the results of the accuracy QA/QC control are given in Table 3.

**Table 3:** Analytical limits for 7 nutritional minerals.

Metals	Concentration range (µg/L)	LOD (µg/L)	LOQ (µg/L)	Ranging calibration curve (µg/L)
Ca	4500–87837*	0.54	1.62	0 -60000
Cu	0.33–57993	0.31	0.93	0–1200
F	27–1553	25	75	0–10000
Mg	1512–19326	1.3	3.9	0 -60000
Mn	0.05–0.05	0.059	0.198	0–1200
Na	1230–161000	0.051	0.153	0 -60000
Zn	0.29–307	0.27	0.81	0–1200

*For values higher than the calibration range, dilution is done.

### Checking the quality index of bottled water nutrients

To determine the BWNQI index and evaluate the contribution of bottled water in the absorption of nutritional minerals 5 factors including: 1. the concentration of nutrient minerals in bottled water; 2. the standard level of nutrient minerals in bottled water; 3. the recommended amount nutritional minerals; 4. the amount of bottled water consumption in different age-sex groups; and 5. age-sex distribution of the population were used. Table 4 shows the amount of bottled water consumption and recommended minerals required by the body in different age-sex groups.

**Table 4:** The amount of bottled water consumption and recommended minerals required by the body in different age-sex groups^51–54^.

Age-sex groups	Recommended level of minerals (mg/d)							Bottled water consumption (L/d)
Infants	Ca	Cu	F	Mg	Mn	Na	Zn	–
0–6 months	350	0.2	–	31	–	120	2.8	0.448
7–12 months	400	0.22	0.5	54	0.6	370	4.1	0.530
Children	–	–	–	–	–	–	–	–
1–3 years	500	0.34	0.5	60	1.2	1000	4.1	0.424
4–6 years	600	0.44	1	76	1.5	1200	4.8	0.545
7–9 years	700	0.7	1	100	1.6	1300	5.6	0.607
Men	–	–	–	–	–	–	–	–
10–18 years	1300	0.9	2.5	220	1.6	1500	7.2	0.907
19–50 years	1000	0.9	3	220	1.8	1500	4.9	1.386
51–65 years	1300	0.9	3	220	1.8	1300	4.9	1.497
> 65	1300	0.9	3	190	1.8	1200	4.9	1.402
Women	–	–	–	–	–	–	–	–
10–18 years	1300	0.9	2.5	230	1.2	1500	8.6	0.907
19–50 years	1000	0.9	4	260	2.3	1500	7	1.576
51–65 years	1000	0.9	4	260	2.3	1300	7	1.568
> 65	1300	0.9	4	224	2.3	1200	7	1.518

Also, the maximum desirable and weighted factor of nutritional mineral ions in bottled water according to the bottled water standard in Iran^55^ is given in Table 5.

**Table 5:** The maximum desirable and weighted factor of nutritional mineral ions in bottled water in Iran.

Nutritional mineral	Maximum desirable (mg/L)	Weighted factor
Ca	80	0.25
Cu	1	0.054
F	1	0.274
Mg	30	0.257
Mn	0.1	0.032
Na	200	0.062
Zn	3	0.071

The following formulas were used to determine the BWNQI index^3^.

1 $$Fij = \frac{{{\text{Ci}} \times {\text{Vj}}}}{{{\text{Aij}}}} \times 100$$

where Fij (%) is the contribution of bottled water in the absorption of nutrients in different age groups, Ci is nutrient concentration (mg/L), Vj is volume of bottled water consumption in different age groups (L) and Aij is the recommended amount of nutrients in different age groups (mg/d).

2 $$Fi = \frac{{\mathop \sum \nolimits_{i = 1,j = 1}^{i = m,j = n} FijPj}}{{\mathop \sum \nolimits_{j = 1}^{j = n} Pj}}\quad i = 1,2, \ldots ,m;\quad j = 1,2, \ldots ,n$$

where, Fi (%) is the contribution of bottled water in nutrient absorption in the total population, Pj is population in different age groups, m is number of nutrient minerals and n is number of age-sex groups.

3 $$Fi,u = \frac{{\mathop \sum \nolimits_{i = 1,j = 1}^{i = m,j = n} \frac{BLi \times Vj \times Pj}{{Aij}}}}{{\mathop \sum \nolimits_{i = 1,j = 1}^{i = m,j = n = n} Pj}} \times 100 \quad i = 1,2, \ldots ,m;\quad j = 1,2, \ldots ,n$$

where Fi, u (%) is the maximum contribution optimal nutrient absorption of minerals in bottled water and BLi is the optimum nutrient concentration (mg/L).

Since manganese, copper, and fluoride ions have adverse effects, the following SIi formula was used to calculate the absorption of these ions.

4 $$SIi = \left( {1 - \left( {\frac{BLi - Ci}{{BLi}}} \right)^{{1 + \left( {Fi,u/\alpha } \right)}} } \right) \times 100$$

For Ci ≤ *BLi*

Where SIi is sub-index of nutritional minerals other than sodium element (unit less) and α is SIi constant and equal to 25.

5 $$SIi = \left( {1 - \left( {\frac{Ci - SVi}{{SVi}}} \right)^{{1 + \left( {\frac{\alpha }{F}`i,u} \right)}} } \right) \times 100$$

For SVi $$< {\text{Ci}} < 2 \times SVi$$

where SVi is the optimum concentration of nutrient mineral at the standard level (mg/L), F`(%) is maximum optimal contribution of bottled water in the recommended adsorption of minerals based on the maximum desired element concentration.

For nutrient concentrations higher than BLi, the SIi 100 was considered. For concentrations higher than 2 times that of SVi, SIi 0 was considered.

6 $$SINa = \left( {1 - \left( {\frac{CNa - BLNa}{{BLNa}}} \right)^{{1 + \left( {\alpha /FNa,u} \right)}} } \right) \times 100$$

For 200 mg/L $$< {\text{Na}} < 400\,{\text{mg}}/{\text{L}}$$

The above equation was used to calculate SINa at concentrations of 200–400 mg/L. For concentrations below 200 and above 400 mg/L, SINa was considered 100 and 0, respectively.

7 $$BWNQI = \frac{{\mathop \sum \nolimits_{{{\text{i}} = 1}}^{{\text{m}}} {\text{Wi SI}}_{{\text{i}}}^{\upbeta } }}{{\mathop \sum \nolimits_{{{\text{i}} = 1}}^{{\text{m}}} {\text{Wi}}}}$$

where Wi is nutritional weight factor, and β is BWNQI constant and equal to 0.9.

The BWNQI index classifies bottled water quality into 5 groups as follows: 80–100 (excellent), 70–79.9 (good), 60–69.9 (fair), 45–59.9 (marginal) and 0–44.9 (poor).

### Sensitivity analysis

In the sensitivity analysis, the effect of each input dietary element on the BWNQI score was considered. Each parameter was removed separately from BWNQI and then its effect on the original BWNQI score was measured.
